# Supplementary material for: Test/Retest Reliability and Validity of Remote vs. In-Person Anthropometric and Physical Performance Assessments in Cancer Survivors and Supportive Partners
Source: Cancers (Basel). 2022 Feb 21;14(4):1075. doi: 10.3390/cancers14041075 (PMC8869803; doi:10.3390/cancers14041075)
Supplement: Supplementary file 1 [file cancers-14-01075-s001.zip › cancers-1587841-supplementary.pdf]

Test/Retest Reliability and Validity of Remote vs. In-Person Anthropometric and Physical Performance Assessments in Cancer Survivors and Supportive Partners

Teri W. Hoenemeyer, William W. Walker. Cole, Robert A. Oster, Dorothy W. Pekmezi, Andrea Pye and Wendy Demark-Wahnefried

Table S1. Comparison of anthropometric and physical performance data obtained between two sequential virtual assessments (reliability) for DUET survivors.

Measurement	Virtual 1 (V1) Mean (SD)	Virtual 2 (V2) Mean (SD)	ICC and 95% CI (V1 vs V2)*	Mean difference (V1-V2)	Limits of Agreement (V1-V2)	p-value
Weight (kg) [n=35]	84.0 (20.1)	85.1 (17.7)	0.94 (0.88, 0.97)	-1.0 (7.0)	-14.7, 12.7	0.39
Waist Circumference (cm) [n=37]	106.5 (15.7)	106.2 (15.8)	0.99 (0.97, 0.99)	0.3 (2.6)	-4.8, 5.4	0.45
30 sec Chair stand (reps) [n=37]	10.2 (2.8)	11.1 (3.6)	0.77 (0.59, 0.87)	-0.9 (2.3)	-5.4, 3.6	0.02
8′ Get Up & Go (secs to 10^{ths}) [n=37]	7.9 (2.4)	7.5 (1.7)	0.66 (0.42, 0.81)	0.4 (1.8)	-3.2, 4.0	0.19
8′ Walk (secs to 10^{ths}) [n=37]	2.2 (0.5)	2.2 (0.5)	0.49 (0.19, 0.70)	-0.0 (0.5)	-1.0, 0.9	0.80
Sit & Reach (cm) [n=36]	-0.8 (3.9)	-0.3 (3.4)	0.85 (0.72, 0.92)	-0.5 (2.1)	-4.6, 3.5	0.13
Backscratch (cm) [n=35]	-3.8 (3.2)	-3.6 (3.0)	0.96 (0.91, 0.98)	-0.2 (1.0)	-2.1, 1.7	0.19
2-min step test (# steps) [n=37]	78.2 (25.5)	81.8 (24.0)	0.92 (0.85, 0.96)	-3.7 (9.7)	-22.8, 15.4	0.028

*ICC=intra-class correlation coefficient, CI=confidence interval.

Table S2. Comparison of the average of virtual assessments with in-person measures (validity) for DUET survivors.

Measures	(V1+V2)/2 Mean (SD)	In-person Mean (SD)	ICC and 95% CI (average virtual vs in-person)*	Mean difference (Average Virtual vs In-person)	Limits of Agreement (Average Virtual vs In-person)	p-value
Weight (kg) [n=36]	84.5 (18.6)	84.7 (17.6)	0.98 (0.96, 0.99)	-0.2 (3.6)	-7.3, 7.0	0.79
Waist Circumference (cm) [n=36]	106.3 (15.7)	102.0 (15.5)	0.96 (0.92, 0.98)	4.3 (4.4)	-4.2, 12.9	<0.001
30 sec Chair stand (reps) [n=37]	10.7 (3.0)	11.4 (3.6)	0.89 (0.78, 0.94)	-0.7 (1.7)	-4.0, 2.5	0.011
8′ Get Up & Go (secs to 10ths) [n=37]	7.7 (1.9)	7.6 (1.5)	0.80 (0.64, 0.89)	0.1 (1.1)	-2.0, 2.3	0.47
8′ Walk (secs to 10ths) [n=37]	2.2 (0.4)	2.1 (0.4)	0.66 (0.42, 0.81)	0.1 (0.3)	-0.6, 0.7	0.19
Sit & Reach (cm) [n=37]	-0.6 (3.5)	-0.4 (3.1)	0.93 (0.85, 0.96)	-0.1 (1.3)	-2.7, 2.5	0.54
Backscratch (cm) [n=37]	-3.7 (3.1)	-3.2 (3.1)	0.96 (0.93, 0.98)	-0.5 (0.8)	-2.1, 1.2	0.002
2-min step test (# steps) [n=37]	80.0 (24.3)	83.3 (28.2)	0.86 (0.73, 0.92)	-3.3 (14.5)	-31.7, 25.1	0.18

*CI=confidence interval.

Table S3. Comparison of anthropometric and physical performance data obtained between two sequential virtual assessments (reliability) for DUET partners.

Measures	Virtual 1 (V1) Mean (SD)	Virtual 2 (V2) Mean (SD)	ICC and 95% CI (V1 vs V2)*	Mean difference (V1-V2)	Limits of Agreement (V1 - V2)	p-value
Weight (kg) [n=36]	88.4 (21.9)	89.7 (19.7)	0.93 (0.86, 0.96)	-1.4 (8.1)	-17.2, 14.4	0.32
Waist Circumference (cm) [n=36]	109.4 (14.2)	108.7 (14.5)	0.98 (0.96, 0.99)	0.7 (3.0)	-5.1, 6.6	0.15
30 sec Chair stand (reps) [n=37]	11.2 (3.1)	12.8 (3.2)	0.82 (0.68, 0.90)	-1.7 (1.9)	-5.3, 2.0	<0.001
8′ Get Up & Go (secs to 10ths) [n=37]	7.7 (1.8)	7.2 (1.8)	0.87 (0.76, 0.93)	0.5 (0.9)	-1.2, 2.2	<0.001
8′ Walk (secs to 10ths) [n=37]	2.2 (0.5)	2.1 (0.4)	0.46 (0.15, 0.68)	0.1 (0.5)	-0.9, 1.0	0.36
Sit & Reach (cm) [n=37]	-0.6 (3.2)	-0.2 (3.1)	0.88 (0.77, 0.93)	-0.3 (1.6)	-3.4, 2.7	0.22
Backscratch (cm) [n=37]	-3.1 (3.5)	-3.3 (3.7)	0.86 (0.73, 0.92)	0.2 (1.9)	-3.6, 4.0	0.56
2-min step test (# steps) [n=37]	76.4 (21.1)	82.9 (21.8)	0.80 (0.64, 0.89)	-6.5 (13.5)	-32.9, 19.9	0.006

*ICC=intra-class correlation coefficient, CI=confidence interval.

Table S4. Comparison of the average of virtual assessments with in-person measures (validity) for DUET partners.

Measures	(V1+V2)/2 Mean (SD)	In-person Mean (SD)	ICC and 95% CI (average virtual vs in-person)*	Mean difference (Average Virtual vs In-person)	Limits of Agreement (Average Virtual vs In-person)	p-value
Weight (kg) [n=36]	89.0 (20.4)	89.6 (19.4)	0.98 (0.96, 0.99)	-0.6 (4.2)	-8.8, 7.7	0.42
Waist Circumference (cm) [n=36]	109.1 (14.3)	105.3 (15.6)	0.95 (0.90, 0.97)	3.8 (4.8)	-5.6, 13.2	<0.001
30 sec Chair stand (reps) [n=37]	12.0 (3.0)	12.7 (2.8)	0.84 (0.70, 0.91)	-0.7 (1.7)	-4.0, 2.6	0.016
8′ Get Up & Go (secs to 10^{ths}s) [n=37]	7.5 (1.6)	7.4 (1.3)	0.79 (0.62, 0.89)	0.1 (1.0)	-1.8, 2.0	0.51
8′ Walk (secs to 10^{ths}s) [n=37]	2.2 (0.4)	2.2 (0.4)	0.66 (0.41, 0.80)	-0.0 (0.3)	-0.7, 0.7	0.99
Sit & Reach (cm) [n=37]	-0.4 (3.0)	0.4 (3.2)	0.86 (0.74, 0.93)	-0.8 (1.6)	-4.0, 2.4	0.005
Backscratch (cm) [n=37]	-3.2 (3.5)	-2.9 (3.3)	0.94 (0.88, 0.97)	-0.3 (1.2)	-2.6, 2.1	0.17
2-min step test (# steps) [n=37]	79.6 (20.4)	87.3 (23.7)	0.84 (0.69, 0.91)	-7.6 (13.2)	-33.5, 18.2	0.001

*CI=confidence interval.

Supplementary File S5: Virtual Visit Script

[**Set-Up:** prior to assessment appointment, participant/partner should receive a package of supplies and a confirmation email with instruction video and Zoom® app. A phone call reminder 2 days prior to appointment will confirm that supplies and email were received, the Zoom® app has been downloaded and participant or partner has the camera equipment necessary for assessment.]

Introduction

ASSESSOR*: Hi Mr. /Ms. (Name). Thank you for agreeing to participate in this assessment. I am* [Your name] *and I will be conducting your virtual assessment today. Before we get started, I want to confirm that your partner is there with you, and you have your supplies ready.*

[If **YES**, greet partner, thank him/her, and remind partner that he or she will be assisting with tests.]

[If **NO**, reschedule the assessment.]

ASSESSOR: *I want to begin by asking if you were able to view the video about what you can expect today that was emailed to you.*

[If **YES**, ask if there are any questions.}

[If **NO**, ask participant/partner to log off the call, watch the video and rejoin the Zoom call in 30 minutes.]

ASSESSOR: Before we start, may I have permission from you to video tape this session?

[If **YES**, start recording.]

[If **NO**, explain that the recording will only be used to verify timed scores and then deleted. If still, "NO", make a note and proceed without recording.]

Anthropometric measures

1) Weight

[**SET-UP**: have participant remove shoes / heavy clothing and empty pockets. Camera should be angled so that scale face can be seen. If the camera needs to be closer, it is held by partner. If scale is digital, you will need to instruct the participant to tap the scale to activate it. Have participant place the scale on a solid, flat surface avoiding carpet.] Camera held by participant. You do not want the weight of a laptop coming up on their scale.

ASSESSOR: *Before we begin, let's test the camera view while you are holding so that I can see the reading. If the camera needs to be closer, [Partner's name], hold it while kneeling down. I will let you know when we are ready to begin.*

[Once view is clear, ask participant to tap scale to activate it, wait for it to zero out, and then step on it. Repeat procedure and record average weight.]

2) Waist circumference

[**SET-UP**: have participant get white and red ribbons and black marker from supplies package. Camera should be angled so that a front view of participant's mid-section can be seen. Camera should be placed on table or chair so that partner can assist with measurement. The angle should be close enough so that assessor can see correct ribbon placement.]

ASSESSOR: *Next, we'll take a waist measurement. You will need to get the red and white ribbons labeled with your name and the marker. We will start with the red ribbon.* [Partner's name] *will need to help with this measurement.*

[Participant's name] *lift your shirt up, hold it with one hand while you place the taped end of the ribbon over your belly button with the other hand. Hold this in place with your finger while.*

[Partner's name], *hold the rest of the ribbon and walk around [Participant's name] so that the ribbon wraps around his/her waist. Make sure that the ribbon is not twisted or caught on any clothing. Then, place the loose end of the ribbon over the taped end and hold both with your finger - the same way as if you were using a tape measure instead of a ribbon.*

[Participant's name], turn around so that I can see the ribbon on your back to make sure it is flat and not twisted.

[Ask participant to take a deep breath in and then breathe all the way out; ask the partner to mark the ribbon with the black marker where the ribbon crosses the taped end and set it aside. Repeat procedure with white ribbon. Remind the participant and partner to return the ribbons in the pre-paid postage envelope along with other returned supplies.]

Physical Performance - Balance test

[**SET-UP**: instruct the participant to stand in a corner or a doorway for safety purposes. Camera can be placed on the floor or on a table chair directly across from participant and angled to capture feet to shoulders in frame. This allows partner to spot participant if balance is a problem. Or partner can hold camera and capture participant's feet to shoulders in frame. This test will be done without shoes.]

ASSESSOR: *To begin, I will ask you to do three different stances and time them for 10 seconds. The first stance is to stand with your feet shoulder width apart. You may hold the wall to get your balance and when I say, "ready, set, GO," let go and hold it until I say stop.*

[Instruct the participant when to start after confirming that you have a feet-to-shoulder view of the participant. Record score as "0" if stance is not held for 10 seconds or "1" if held for 10 seconds.]

ASSESSOR: *The second stance is "semi-tandem" stance. You may hold onto the wall to balance yourself. Place the heel of your dominant foot into the arch of the other foot so that they are touching, and one foot is slightly in front of the other. When*

I say, "ready, set, GO," let go of the walk and hold this stance until I say stop. Reach out to the wall and steady yourself and break your stance.

[If this is a baseline test, encourage the participant to experiment with which foot feels comfortable in the forward, or dominant position. Instruct the participant when to start after confirming that foot placement is correct, and you have a foot-to-shoulder view of the participant. Record score as "0" if stance is not held for 10 seconds or "1" if held for 10 seconds.]

ASSESSOR: *Next is the full tandem stance. You may hold onto the wall until I tell you to go. For this test, place your dominant foot fully in front of the other foot, heel to toe. You will hold this until I say stop. Once I say, go, let go of the wall and the test begins. If you feel you will fall, hold onto the wall, or place your foot normally. I will then stop the test.*

[Instruct the participant when to start after confirming that foot placement is correct, and you have a foot-to-shoulder view of the participant. Record score as "0" if stance is not held for 10 seconds or "1" if held.]

Physical Performance Measures - SFT

1) **30 Second Chair Stand**

[**SET-UP**: Instruct participant or partner to get the chair they plan to use and ensure chair (18" un-cushioned chair preferred) is placed against the wall for safety. Encourage the participant to place a dish towel on the back to avoid marring the wall). Camera can be placed on floor at a 90-degree angle with a direct view of participant fully seated and fully standing. Camera can also be held by partner.]

ASSESSOR: [Participant's name], *remain seated in the chair, cross your arms, and place your hands on opposite shoulders. Once you are ready, I will say, "OK, Ready, Set, GO!" When you hear "go" you will begin to stand up fully and then sit back down, making sure that you keep your arms crossed at shoulder level. You will repeat this motion, doing as many as you can until I say "Stop."*

[Instruct participant when to start and stop at 30 seconds; record the number of complete stand-to-sit motions].

2) **Chair Sit-and-reach**

[**SET-UP**: instruct partner to place the camera on floor at a 90-degree angle up to see participant in both seating and standing positions.]

ASSESSOR: *Next, we will move into the Chair sit-and-reach.* [Participant's name], *have a seat in the chair.* [Partner's name], *for this test you will need the tape measure.*

[Participant's name], *starting from a seated position towards the edge of the chair, you will extend one leg straight, leaving the other bent at a right angle or 90 degrees for stability. Place your hands on top of one another* [demonstrate on screen for participant to see] *and reach as far as you can towards the toes of your extended leg. Make sure the toes are pointed straight towards the ceiling. When you are ready, reach as far as you can and try to touch or pass your toes, making sure not to bend your knee. If your knee starts to bend, stop there while it's measured.*

[Partner's name], *kneel by* [Participant's name] *with the tape measure and measure the distance from their longest finger to their big toe and tell me that measurement in inches.*

[Repeat this test on the other leg and record the best measurement of the two. Not touching = negative score, fingers touching = 0, overlapping = positive score.]

3) **Back Scratch**

[**SET UP**: instruct the partner to place the camera on table, chair, or counter, angled to see participant's back and observe the partner's measurements – similar to the angle for waist circumference. Direct the participant to start on arm that feels the most flexible. Inform partner that he/she will need measuring tape.

ASSESSOR: *Next, I will ask you to do the back scratch test.*

[Participant's name], I will ask you to *stand and reach with* [left/right] *arm straight up above your head and then reach behind you head and down your back. With the other arm reach up your back and try to touch your fingers to the fingers on*

the other hand. Do not push past discomfort as we do not want injuries, but reach as far as you are able, attempting to touch your fingers.

[Partner's name], *take the tape measure and measure the distance between the two middle fingers and tell me that number in inches.*

[Repeat this test on the other arm and record the best measurement of the two. Not touching = negative score, fingers touching = 0, overlapping = positive score.]

---

4) 8″ Get Up and Go

ASSESSOR*: Before we begin with walking tests, were you able to clear a 12-foot space?*
[If YES, begin the Set-up procedure.]

[If NO, ask participant/partner to find a space and clear it.]

[**SET-UP**: Instruct the partner to place the 8′ yellow cord, stickers, and orange cones near the chair. Instruct the partner to place camera on floor far enough away so that participant in the seated and standing positions and the 8′ end point orange cone can be viewed in one frame. Typically, the camera is located behind the cone, facing the chair and participant. Camera can also be held by partner using same frame. Participant will wear shoes for this test.)
ASSESSOR*: This test is the 8′ foot get-up-and-go. [Participant's name], you need to sit in the chair and place your feet on the floor positioned as if you are about to stand up.* [Partner's name], *take one sticker and place it under* the top of [Participant's name] big *toe on either the left or right foot.*
Next, place one end of the 8′ foot cord on top of that sticker and while [Participant's name] *holds the cord in place, pull it straight out from the chair. Where the black mark is on the cord, place a sticker on the floor. Then, place the orange cone so that the edge farthest from the chair covers the sticker.*
This is a timed test, so perform it as quickly and safely as possible, without running. When I say "ready, set, go," you will stand up keeping your arms crossed, release them as you walk to the cone, go around the cone, return to your chair and sit back down.
[For accurate timed measurement, view the video and record time stamp.]

---

5) Timed 8′ Walk

[**SET-UP**: have partner move cone on sticker to the side and place the other cone directly opposite to form an 8′ "finish line". Camera is positioned on floor far enough away to frame the 8-foot distance finish line (orange stickers), the starting point, and participant's feet. Camera can also be held by partner with view of entire distance in frame. Instruct the partner to remove the chair since this test will be started from a standing position from the sticker that was placed by the chair. Participant will wear shoes for this test.]
ASSESSOR: *This next test is similar to the last one, except you will start from a standing position and go through an 8′ finish line marked by the orange cones. When I say "ready, set, go" the timer will start, you will walk from your starting place where your toe is on the sticker and walk through the finish line marked*
on either side by the cones.
[For accurate timed measurement, view the video and record time stamp.]

6) 2-minute Step Test

[**SET-UP**: ask participant to stand in a corner or doorway for safety. Partner will need to have a sticker and tape measure nearby. Camera can be placed on floor angled up to view participant's waist to feet to ensure correct ratio measurement are done by partner. For step test, camera can be held by partner and angled so that wall sticker and full view of participants are in the same frame. The test should be performed without participant's hands on wall or door. Test may be paused should participant need to rest. However, timer is not stopped for pauses.]

ASSESSOR: *This will be our final test today. For this test, I will instruct* [Partner's name] *on how to do the hip-to-knee measurement. [Participant's name], lift your knee and on the side of your leg feel where your leg bone moves at your hip joint. Put your finger here.* [Partner's name], *take the tape measure and place the end at his/her finger and measure to the top of his/her knee cap. What is that number? (i.e., distance from tip of trochanter to tip of patella) Continue holding the tape there while I make a calculation.*

[Calculation: divide this number by 2, e.g., if it is 19, then 9.5 inches.]

[Partner's name], *while still holding your tape in place, place a sticker on this midpoint. Now, measure the distance from that sticker to the floor. What is that distance?*

[Instruct the partner to use measure this distance from the floor up a wall and mark it with a sticker. Also, record this distance in REDCap so that the exact same value can be used for follow-up assessments.]
ASSESSOR: [Participant's name], *now you will march in place for 2 minutes, making sure that your knees come up to the sticker on the wall, no higher, no lower. Do not talk during this test as it may lower your score. When I say "ready, set, go," you will begin. You may stop if you need to catch your breath or rest. However, the timer will not stop. I will say "stop" when the test is over.*

[Count every other step for score; test is stopped at 2 minutes and results are recorded.]

CLOSING

ASSESSOR: *Now, the assessment is completed. You can rest and have a drink of water if needed. Our study team appreciates your flexibility and willingness to participate in this assessment and our study. Be sure to contact us if you have any questions or concerns. We'll see you again* [next assessment].

[Assure that participant/partner can disconnect. End video and save to secured drive. End call.]
